# Longitudinal validation of the Maudsley 3-item visual analogue scale (M3VAS): a new, brief, patient-reported outcome measure of depression

**DOI:** 10.1192/bjo.2025.10932

**Published:** 2025-12-22

**Authors:** Daniel Silman, Maria Elena Middag, Anthony J. Cleare, Allan H. Young, Rebecca Strawbridge

**Affiliations:** Centre for Affective Disorders, Department of Psychological Medicine, Institute of Psychiatry, Psychology & Neuroscience, King’s College London, London, UK; South London and Maudsley NHS Foundation Trust, Bethlem Royal Hospital, Beckenham, UK; Division of Psychiatry, Department of Brain Sciences, Imperial College, London, UK

**Keywords:** Antidepressants, bipolar type I or II disorders, depressive disorders, diagnosis and classification, mood stabilisers

## Abstract

**Background:**

The Maudsley 3-item visual analogue scale (M3VAS) was developed as a novel and intuitive patient-reported measure for depression, focusing on core symptoms and suicidality.

**Aims:**

To evaluate the longitudinal validity of M3VAS for capturing symptom change over time.

**Method:**

Both M3VAS and the Patient Health Questionnaire (PHQ-9, as reference standard) were administered in an observational study (RHAPSODY, no. NCT04939818) at weeks 0, 2 and 4 to both depressed patients (*n* = 50) and matched controls (*n* = 24). We serially tested factor structure, internal consistency and convergence (correlation) over time, assessing responsiveness by both correlation of change in score and effect of time across scales (analysis of variance and effect size).

**Results:**

M3VAS exhibited strong factor loadings and high item interrelatedness (Cronbach’s alpha 0.78–0.83) at all time points. Total scores correlated strongly with PHQ-9 at each time point (*r* > 0.8, *P* < 0.001). Correlation of score change over the study period (*r* = 0.65, *P* < 0.001) also confirmed responsiveness. In the depressed group, an effect of time on score was seen for both M3VAS (*F* = 4.942, *P* = 0.010) and PHQ-9 (*F* = 12.505, *P* < 0.001), with standard response mean (Cohen’s *d*) of 0.58 and 0.74, respectively. No effect of time was seen in the control group.

**Conclusions:**

Following previous cross-sectional validation against the Quick Inventory of Depressive Symptomatology–Self-report, this present study demonstrated appropriate longitudinal measurement properties for M3VAS as a measure of depression, including responsiveness. Evaluating the ability of M3VAS to discern responses with a variety of treatments is a key future goal.

The limitations of gold-standard approaches to clinical measurement of depression symptoms are increasingly being emphasised.^1–5^ Major depressive disorder (MDD) can be highly heterogenous beyond the requirement for significant and pervasive low mood and/or anhedonia as the two core symptoms.^6^ Standard, full-length outcome measures with conventional sum-scoring could place undue emphasis on symptoms that are less universally clinically relevant – the Hamilton Depression Rating Scale (HAM-D),^7^ for example, includes symptoms not featured in modern diagnostic criteria for MDD (e.g. gastrointestinal) and those potentially reflecting medication side-effects (e.g. somatic symptoms, agitation). These limitations, contributing to the lack of a coherent singular underlying measurement construct,^1,8^ may be hampering sensitivity to capture treatment effects in clinical trials.^9^ Recent secondary data analyses have shown a noteworthy trend of increased antidepressant separation from placebo utilising singular measurement of mood^10^ or shorter subscales.^11,12^ Creating shorter depression outcome measures by concentrating on symptoms that have higher clinical relevance and specificity to depression therefore presents an attractive option. Self-report measures that are rapidly and intuitively completed have the added potential as digital measures to contribute to enhanced patient self-monitoring and ecological momentary assessment – approaches which, per se, could enrich understanding of affective dynamics and predictors of treatment course in depression.^13^ While a range of such brief/digital mood measurements exist, few have been subject to substantive validation,^14^ especially in regard to being considered as a clinical trial end-point measure.

To this end, we developed the Maudsley 3-item visual analogue scale (M3VAS), a novel instrument that proposes the capture of brief and sensitive measurement of the two core depressive symptoms (low mood and anhedonia), alongside suicidality, due to its high clinical pertinence. A VAS response style was favoured as having demonstrated higher degrees of resolution, and consequently greater sensitivity to change over time, than standard Likert scales for physical disorders.^15,16^ Selection of appropriate anchor points on the scale may also allow for more intuitive capture of a global level of subjective difficulty (e.g. ‘not at all’ versus ‘extremely’ depressed) over items in gold-standard Likert scales that have been suggested to be limited by exclusive focus on symptom frequency, which may impede patients’ conveying meaningful change – as observers have noted in regard to Patient Health Questionnaire 9 (PHQ-9).^17,18^ Adoption of VAS for depression has undergone minimal iteration since first employed by Zealley and Aitken in 1969^19^ – exclusively focusing on singular mood assessment, which is typically contemporaneous.^20,21^ Although reasonable validity has been shown,^22^ we hypothesised that this could be optimised in M3VAS, which covers pervasiveness over the previous 2 weeks, with item combination enriching diagnostic and clinical utility while maintaining simplicity.

Within clinical trial populations we have previously demonstrated the cross-sectional validity of M3VAS showing a favourable single-factor structure, internal consistency and strong convergence with the Quick Inventory of Depressive Symptomatology–Self-report (QIDS-SR-16^23^). As a further step in validating M3VAS for ongoing monitoring of depression severity, we now examine its longitudinal measurement properties, this time against PHQ-9 during a recent non-interventional clinical study over 4 weeks (RHAPSODY^24^). Specifically, the objectives were to:

- determine stability of the internal structure (factorial validity and internal consistency) of M3VAS on repeated measurement;
- determine convergent validity between M3VAS and PHQ-9 scores;
- determine responsiveness of M3VAS against PHQ-9 by both convergence of score changes over time and evaluating the effect of time on scores.

## Method

A protocol for this secondary analysis was developed prior to data access (published on a preprint server^25^). Longitudinal assessment of the scale’s internal structure was added post hoc at the discretion of the investigators (see Supplementary Table 1 for the full list of amendments since the original protocol).

### Design

The RHAPSODY study^24^ was an observational, longitudinal study that examined speech phenotyping for remote evaluation of neurodegenerative and psychiatric conditions. Self-reported measures of depressive symptoms were collected at baseline (w0) and at 2 (w2) and 4 weeks (w4), in addition to data from verbal cognitive and speech tasks. The authors assert that all procedures contributing to this work comply with the ethical standards of the relevant national and institutional committees on human experimentation, and with the Helsinki Declaration of 1975 as revised in 2013. All study procedures were approved by the Health Research Authority and Health and Care Research Wales (REC reference no. 21/PR/0070). Written informed consent was obtained from all volunteers involved in the study.

From the parent study (which included additional groups with other neurodegenerative conditions), this secondary analysis focuses on two participant groups: patients with a current depressive episode and healthy controls – in whom score change is likely to be smaller, allowing for group comparison including differences in effect of time by depression status, alongside the pooled analyses. Data from 74 participants were included in our analysis which, in reference to COSMIN consensus criteria,^26^ was considered an adequate sample size with which to conduct the principal analyses to meet the study objectives (subgroup analyses that are at risk of bias due to doubtful sample size are highlighted in the Discussion, below).

### Participants

Participants were recruited from July 2021 to June 2022 via local clinical services, research databases and community advertisements. Participants were required to be native English speakers aged 18–85 years in order to participate.

Eligible for the affective disorder group (*n* = 50) were those experiencing a current major depressive episode according to DSM-IV, as assessed by the Mini-International Neuropsychiatric Interview v.5.0;^27^ individuals could have a diagnosis of either bipolar disorder or MDD. Depressive symptoms were required to be of at least moderate severity, as assessed using the Clinical Global Impression scale.^28^ Any treatment status was accepted (and not overseen by the study team).

Unaffected healthy controls were matched to the affective disorder group for gender, age and education levels. Healthy controls were permitted to have mild physical comorbidities (including respiratory, immunological, metabolic or cardiologic conditions) that did not hinder daily functioning. Participants were excluded (all groups) if they had a current substance use disorder; had experienced a stroke within the past 2 years, a transient ischaemic attack or unexplained loss of consciousness within the past 12 months; presented with a current risk of suicide; or lacked the appropriate digital device requirements for participation.

### Measures

All participants concurrently completed the two depression patient-reported outcome measures (PROMs) at w0, w2 and w4. M3VAS^23^ asks participants to rate their experience of low mood, anhedonia and suicidality over the past 2 weeks by making a mark on a 100 mm unmarked line, resulting in a score of between 0 and 300. PHQ-9^29^ is a widely used depression scale that scores each of the 9 DSM-IV diagnostic criteria for depression, from 0 (not at all) to 3 (nearly every day).

### Statistical analysis

In addition to the full-scale score, a subscale of total PHQ-9 scores combining items 1 (anhedonia), 2 (mood) and 9 (suicidality) was calculated to mirror M3VAS (referred to here notionally as ‘PHQ-D’) for directly matched item comparison. The totals of the remaining six items were then combined to assess for any further divergence from the indirect domains of PHQ (referred to here as ‘PHQ-I’).

In addition to summary descriptive statistics (mean, standard deviation) for baseline characteristics and PROM scores at the various time points, the following analyses were conducted according to the study objective.

### Objective 1: internal structure over repeated measurements

Factorial validity: factor analysis was evaluated at all three time points for M3VAS, PHQ-9 and the PHQ-D subscale for comparison. To assess suitability, we required a Kaiser–Meyer–Olkin (KMO) Measure of Sampling Adequacy >0.6 and a significant Bartlett’s Test of Sphericity (*P* < 0.001).^30^ Principal axis factoring was used as the (exploratory) extraction method to evaluate the factor structure of each respective scale. An oblimin rotation, Promax with Kaiser normalisation, was employed to account for potential correlation between the emerging factors. Eigenvalues greater than *λ* = 1 determined the appropriate number of factors to extract.^31^ While only a single factor can be extracted from the 3 item scales, variance explained and factor loadings were explored in considering evidence of unidimensionality (with loadings ≤0.5 considered problematic).

Internal consistency: pairwise correlations between each item were evaluated using Cronbach’s alpha. The test for Cronbach’s alpha generates a value with 1.0 as the maximum outcome, and values closer to 1.0 indicating greater internal validity,^32,33^ interpreted according to the following ranges: *α* ≥ 0.90, excellent; 0.90 > *α* ≥ 0.80, good; 0.80 > *α* ≥ 0.70, acceptable; 0.70 > *α* ≥ 0.60, questionable.^34^ Alpha if item deleted (AID) was considered for each item, with items of good internal consistency producing values lower than Cronbach’s alpha value. Item total correlation (ITC) values were calculated, with those between 0.3 and 0.8 generally indicating good internal consistency.^34^

### Objective 2: convergence validity between M3VAS and PHQ-9 scores

The Pearson correlation coefficient *r* (with corresponding two-tailed *P*-values) was calculated between the M3VAS and PHQ-9 totals at each time point. This was performed for the total sample population (to observe global convergence across the spectrum of symptoms) and between groups for comparison, with *r* > 0.70 considered good criterion validity.^35^ This was repeated between M3VAS and the PHQ-D and PHQ-I subscales (with *P* significance threshold adjusted (0.015) for multiple comparisons, Bonferroni method). Correlation against PHQ-9 is also presented graphically using scatter plots, reporting *R* ^2^ and 95% confidence intervals associated with the line of best fit. Pearson’s correlation was also measured for individual M3VAS items against the three corresponding PHQ-9 items.

As a secondary examination of the sensitivity of M3VAS in detecting mild depressive symptoms, we analysed participants who scored 0 or 1 out of 3 on each PHQ-D item and observed the proportion who scored over 50 on the corresponding M3VAS item.

### Objective 3: responsiveness of M3VAS (against PHQ-9)

As per the criterion approach for assessment of responsiveness,^36^ changes in score on M3VAS over the various intervals (w0–w2, w2–w4, w0–w4) were assessed for correlation (Pearson’s *r*) with corresponding changes on PHQ-9 (as the reference standard, *r* > 0.50 is considered strong^36^) over the same interval. This was also calculated across the total sample and for each group separately, then repeated for the PHQ-D and PHQ-I subscales (*P* significance threshold similarly adjusted (0.015) for multiple comparisons).

To further assess a construct approach for capturing score changes, we anticipated that an effect of time would be observed on M3VAS scores. This was first assessed by repeated-measures analysis of variance (ANOVA), examining the main effect of time on scale scores for the combined sample and each group separately (i.e. depressed and healthy), as well as the interaction effect of time and group.^37^ Time (w0, w2 and w4) represented the within-subjects factor, with group (affective disorder versus healthy controls) the between-subjects factor. Mauchly’s test of sphericity was used to investigate violation of the univariate assumption (requiring *P* > 0.05). Additionally, paired-samples *t*-tests^38^ were used to determine whether there were statistically significant changes in depressive symptoms over various time windows (*P* < 0.025 determined significant, Bonferroni method) according to either measure. Finally, the standardised response means (Cohen’s *d* ^39^) were estimated by dividing the mean change between time points by the standard deviation of the mean change (mean change/s.d. mean change), to provide a coefficient of change (effect size) for each measure, both across the groups and combined.

All analysis was conducted using IBM SPSS Statistics version 27.0 for Windows (IBM Corp., Armonk, NY, USA; see https://www.ibm.com/products/spss-statistics).

## Results

### Population characteristics

From the RHAPSODY data-set (*n* = 173, all clinical groups), exclusions were made based on inappropriate group and incomplete/excess data entries at baseline (w0). A total of 74 participants were included in the data analysis cohort for the groups of interest: affective disorder (*n* = 50, comprising 24 with MDD and 26 with bipolar disorder) and their matched controls (healthy controls, *n* = 24). Of these 74 participants, 69 (93%) subsequently provided complete data on M3VAS and 66 (89%) on PHQ-9 at w2. At w4 this dropped further to 55 (74%) and 53 (72%), respectively. The baseline sample was highly gynocentric, with 13 male participants (17.5%), 60 female (81.1%) and 1 other (1.4%). The majority (*n* = 46, 62%) of participants were of White ethnicity. Groups were matched for gender, age and level of education as per protocol, although there was a significant mean difference in body mass index of 4.33 between the groups (*t* = −2.55, *P* = 0.006) at baseline, as shown in Table 1.

**Table 1:** Participant characteristics and symptom scores

Participant characteristics	Affective disorder group	Healthy controls group	Overall
*N* (baseline)	50	24	74
Female (*n*, %)	40 (80.0)	20 (83.3)	60 (81.1)
Age (mean, s.d.)	37.2, 14.4	36.6, 14.9	37.0, 14.4
Education (years (mean), s.d)	15.5, 2.6	15.4, 2.1	15.5, 2.5
BMI (mean, s.d.)^a^	28.4, 7.8	24.1, 4.2	27.0, 7.1
M3VAS scores (mean, s.d. (*n*))			
Baseline	153.3, 37.2 (50)	18.0, 24.1 (24)	109.5, 72.0 (74)
Week 2	139.9, 51.9 (49)	23.4, 24.1 (20)	106.1, 70.0 (69)
Week 4	121.9, 57.7 (34)	28.5, 30.4 (21)	86.2, 66.9 (55)
PHQ-9 scores (mean, s.d. (*n*))			
Baseline	18.0, 4.0 (50)	2.75, 2.3 (24)	13.1, 8.0 (74)
Week 2	14.6, 4.7 (47)	3.2, 2.7 (19)	11.3, 6.7 (66)
Week 4	13.5, 6.1 (33)	3.0, 2.4 (20)	9.6, 7.2 (55)

BMI, body mass index; M3VAS, Maudsley three-item visual analogue scale for depression; PHQ, Patient Health Questionnaire. Groups were matched for gender, age and level of education as per protocol.

a. There was a significant mean difference in body mass index of 4.33 between groups (*t* = −2.55, *P*= 0.006).

### Internal structure

M3VAS was suitable for factor analysis at all time points, with KMO >0.60 (0.60 at w0, 0.63 at w2 and 0.63 at w4) and a significant Bartlett’s test of sphericity *P* < 0.001 (*χ* ^2^(3) = 145.06, 96.96 and 56.56, respectively). The extracted factor (*λ*1 = 2.263, 2.229 and 2.134, respectively) corresponded to a broadly consistent proportion of the total variance – 75.4, 74.3 and 71.1%, respectively. Factor loadings for the individual items were also consistent across the time points, with suicidality (0.704–0.738) lower than the two core symptoms (low mood 0.910–0.949, anhedonia 0.872–0.931; see Supplementary Table 2). The factor structure for PHQ-D (*λ*1 = 2.219 for w0, 2.295 for w2 and 2.328 for w4) appeared broadly similar in both variance explained (74.0, 76.5 and 77.6%, respectively) and item loading distribution, with suicidality loading slightly higher (0.746–0.823) than for M3VAS.

PHQ-9 demonstrated a single factor at w0 (*λ*1 = 5.913) and w4 (*λ*1 = 5.761), with a corresponding lower proportion of variance explained (65.7 and 64.0%, respectively) than the two short scales, and a 2-factor structure at w2 (*λ*1 = 5.298 [58.9%], *λ*2 = 1.041 [11.6%]), in which suicidality (1.035), psychomotor retardation (0.779) and low mood (0.726) loaded most strongly onto factor 1; and poor sleep (0.956), low energy (0.942) and low appetite (0.770) loaded most strongly onto factor 2 (full data, including factor loadings, are shown in Supplementary Table 2).

Cronbach’s alpha for M3VAS was 0.83 at w0 and w2, and 0.78 at w4. Alpha values appeared similar to those for M3VAS for the comparable PHQ-D scale (0.82, 0.85 and 0.84, respectively), being higher for PHQ-9 (0.93, 0.91 and 0.93, respectively). For both the shorter scales, ITCs were consistently higher (range 0.72–0.88) and AID lower (range 0.51–0.73) for the two core symptoms than for suicidality (ITC 0.48–0.64, AID 0.85–0.95; see Supplementary Table 3).

### Convergent validity

**Fig. 1:**
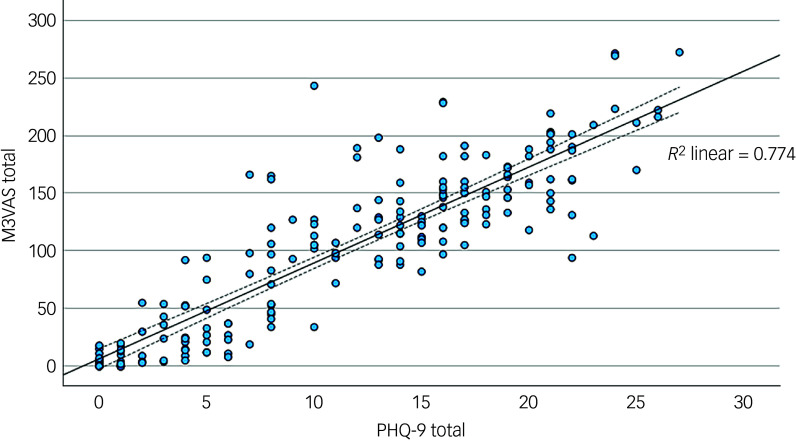
Scatter plot correlating total outcome measure scores for M3VAS and PHQ-9. All time point data were pooled (i.e. to include paired data). *R* ^2^, coefficient of determination for fit line (solid), with dashed lines indicating 95% confidence interval. M3VAS, Maudsley three-item visual analogue scale for depression; PHQ-9, Patient Health Questionnaire 9.

The mean and standard deviation of the total scores for M3VAS and PHQ-9 across groups and time points are detailed in Table 1. The M3VAS total scores correlated strongly with those of PHQ-9 at each time point (*r* > 0.8), being highest at baseline (*r* = 0.91). Convergence did not appreciably increase against only the directly relevant items of PHQ-D (0.88 *v*. 0.87 overall; see Table 2. By group, there appeared to be greater separation between M3VAS convergence with PHQ-D (*r* = 0.71 in the affective disorder group and 0.61 in healthy controls overall) and PHQ-I (*r* = 0.59 in the affective disorder group and 0.54 in healthy controls), with correlation against the full PHQ-9 consistently stronger in the affective disorder group than in controls. M3VAS and PHQ-9 correlation at all time points combined is presented visually in Fig. 1.

**Table 2:** Convergent validity and responsiveness of M3VAS against PHQ-9 and its derivatives

Pearson’s correlation *R* (M3VAS/PHQ)									
Raw scores					Score change				
		Group					Group		
Time point	Comparator	Affective disorder	Healthy controls	Overall	Interval	Comparator	Affective disorder	Healthy controls	Overall
w0	PHQ-9	0.57**	0.55*	0.91**	w0–w2	PHQ-9	0.45**	0.36	0.47**
	PHQ-D	0.58**	0.61**	0.89**		PHQ-D	0.58**	0.19	0.58**
	PHQ-I	0.45**	0.46	0.88**		PHQ-I	0.25*	0.33	0.29
w2	PHQ-9	0.65**	0.61*	0.86**	w2–w4	PHQ-9	0.62**	0.64*	0.63**
	PHQ-D	0.74**	0.55*	0.86**		PHQ-D	0.73**	0.68*	0.73**
	PHQ-I	0.49**	0.55**	0.80**		PHQ-I	0.44*	0.50	0.46**
w4	PHQ-9	0.76**	0.68**	0.87**	w0–w4	PHQ-9	0.62**	0.46	0.65**
	PHQ-D	0.75**	0.66**	0.85**		PHQ-D	0.58**	0.54	0.62**
	PHQ-I	0.70**	0.62*	0.84**		PHQ-I	0.59**	0.30	0.59**
All	PHQ-9	0.69**	0.60**	0.88**					
	PHQ-D	0.71**	0.61**	0.87**					
	PHQ-I	0.59**	0.54**	0.84**					

M3VAS, Maudsley three-item visual analogue scale for depression; PHQ-9, Patient Health Questionnaire 9; w0, baseline time point; w2, week 2 time point; PHQ-D contains PHQ-9 items 1, 2 and 9, which match M3VAS symptoms; PHQ-I includes the remaining six PHQ-9 items; w4, week 4 time point; PHQ-9, Pearson’s correlation coefficient between measures at each time point: by group and overall.

*Statistically significant correlation using two-tailed *P* < 0.015 (i.e. alpha of 0.05 was adjusted for multiple comparisons (x3) of M3VAS at each time point; **P < 0.001; interpretation: *r* ≥ 0.7 for cross-sectional and ≥0.5 for score changes indicate strong correlation.^31^

Exploring individual scale item correlation (see Supplementary Table 4), the strongest associations were consistently observed between corresponding items on M3VAS and PHQ-9 (*r* = 0.70–0.86). Weaker relationships were noted between the suicidality items and core-depressive symptoms on the alternate scale (*r* < 0.5). Of those who scored 0 or 1 out of 3 on each PHQ-D item, 30 out of 111 (27%) for low mood, 30/107 (28%) for anhedonia and 6/174 (3.4%) for suicidality rated their severity as >50 on the corresponding M3VAS item across all time points – as an index of potential differing sensitivities to detect mild symptoms.

### Longitudinal correlation of score changes (responsiveness criterion approach)

Pearson’s correlation analyses were then conducted between score changes from baseline to the follow-up time points on M3VAS against PHQ-9 – including by group and for the PHQ subscales (Table 2). Agreement was generally strong and significant between M3VAS and PHQ-9 for the pooled study population, with *r* values ranging from 0.47 to 0.65 (all *P* < 0.001). In this full sample, score changes appeared more closely correlated between the direct items of PHQ-D (0.58 and 0.73) than the indirect PHQ-I (0.29 and 0.46) over the windows between individual study visits (w0–w2 and w2–w4), although this effect disappeared considering the full monitoring period (w0–w4 change). The weakest correlation of score change between M3VAS and PHQ-9 was observed for the healthy controls group over the w0–w2 interval (*R* = 0.36 *v*. 0.45 for the affective disorder group), although this was higher at the w2–w4 interval (0.64 for healthy controls *v*. 0.62 for the affective disorder group).

### Effect of time on scores (responsiveness construct approach)

An effect of time on scores was shown for M3VAS (*F* = 4.94, *P* = 0.010), PHQ-9 (*F* = 12.51, *P* < 0.001) and PHQ-D (*F* = 8.53, *P* < 0.001) on repeated-measures ANOVA in the affective disorder group, but not for any scale in the healthy controls group (Table 3). When the groups were combined, this effect of time was preserved to statistical significance for both PHQ-9 and PHQ-D (although Mauchly’s test of sphericity was not upheld), and was close to the significance threshold for M3VAS (*P* = 0.052). Group status (as the between-subject factor) was confirmed to have significant interaction with the effect of time on score across measures (see Supplementary Table 5 for full details on ANOVA and Mauchly’s test).

**Table 3:** Score changes over time for M3VAS, PHQ-9 and PHQ-D and associated effect sizes and effect of time

	M3VAS					PHQ-9					PHQ-D				
Group	*F*	Interval	w0–w2	w2–w4	w0–w4	*F*	Interval	w0–w2	w2–w4	w0–w4	*F*	Interval	w0–w2	w2–w4	w0–w4
Overall	3.039	MC	9.32	6.96	13.44	10.073^a^	MC	2.46*	1.16	2.40*	6.285^a^	MC	0.97*	0.33	0.87*
	*P* = 0.052	*P*	0.067	0.260	0.031	*P* < 0.001	*P*	<0.001	0.026	<0.001	*p* = 0.004	*P*	<0.001	0.123	0.004
		*d*	0.22	0.16	0.30		*d*	0.56	0.32	0.51		*d*	0.49	0.22	0.42
Affective disorder	4.942	MC	14.63	12.21	26.88*	12.505	MC	3.49*	1.72	3.85*	8.527	MC	1.40*	0.56	1.49*
	*P* = 0.010	*P*	0.036	0.168	0.002	*P* < 0.001	*P*	<0.001	0.026	<0.001	*p* < 0.001	*P*	<0.001	0.080	0.001
		*d*	0.31	0.24	0.58		*d*	0.76	0.41	0.74		*d*	0.64	0.32	0.64
Healthy controls	0.424^a^	MC	−3.70	−1.95	−8.33	0.082	MC	−0.11	0.21	0.00	0.376	MC	−0.11	−0.05	−0.15
	*P* = 0.578	*P*	0.288	0.794	0.268	*P* = 0.922	*P*	0.840	0.678	1.000	*p* = 0.689	*P*	0.429	0.804	0.453
		*d*	−0.25	−0.06	−0.25		*d*	−0.05	0.10	0.00		*d*	−0.19	−0.06	−0.17

M3VAS, Maudsley three-item visual analogue scale for depression; PHQ-9, Patient Health Questionnaire 9; PHQ-D contains PHQ-9 items 1, 2 and 9, which match M3VAS symptoms; *F*, repeated-measures analysis of variance examining effect of time with accompanying *P*-value; w0–w2, baseline to week 2; w2–w4, week 2 to week 4; w0–w4, baseline to week 4; MC, mean change in score over the stated interval; *P*, accompanying *P*-value for associated paired *t*-test; *d*, Cohen’s *d* (standardised response means). Negative values (e.g. in the healthy controls group) indicate score increase.

a. Greenhouse−Geisser correction where sphericity assumption has been violated (see Supplementary Table 5 for Mauchly’s test).

*Above MC denotes significance at *P* < 0.025 to account for multiple comparisons; interpretation: scores >0.5 indicate moderate effect.^32^

In the affective disorder group, paired-samples *t*-tests detected significant score reductions (*P* < 0.025 to account for multiple time point comparisons) across the full study window (w0–w4) on all three scales – M3VAS, PHQ-9 and PHQ-D (Table 3). PHQ-9 appeared to have greater effect size as measured by Cohen’s *d* (*d* = 0.74 *v*. 0.58 for M3VAS) in this group, as well as showing an additional statistically significant score reduction in the first study interval (w0–w2) – as did PHQ-D. No significant changes were detected in any of the measures for the same intervals for the healthy control group. When scores from both groups were pooled, score reductions on M3VAS stopped short of the significance threshold (w0–w4: *t*(54) = 2.22, *P* = 0.031, *d* = 0.30), while both PHQ-9 and PHQ-D continued to detect significant reductions from w0–w2 and w0–w4.

## Discussion

In this study we evaluated the key longitudinal psychometric properties of M3VAS for patient-reported symptoms of depression in a non-interventional setting among a group of 74 participants comprising both symptomatic patients and healthy controls.

When measured serially over the 3 time points in the study (w0, w2 and w4), M3VAS maintained good structural validity as demonstrated by strong factor loadings and high item interrelatedness (Cronbach’s alpha) – replicating those shown previously in a cross-sectional validation.^23^ These findings give further support to the suggestion that M3VAS reliably measures a coherent underlying mood construct – a favourable characteristic given the multifactorial structure observed in other depression scales.^2,40^ However, a larger sample would be needed to confirm longitudinal measurement invariance by confirmatory factor analysis, affirming factorial stability over time. This is a limitation of the current sample and should be incorporated into more substantial evaluation.

There is also some apparent non-linearity in the M3VAS item relationship, given the higher factor loadings and ITCs (and lower AID) for the two core symptoms of depression (low mood and anhedonia) than for the suicidality item. Such findings fit the clinical observation of suicidality having complex underpinnings beyond current depression severity.^41^ Responses on VAS could be inherently different for suicidality than for other symptoms, as suggested by the fact much higher proportions with mild/absent rating on PHQ-9 for low mood (item 2) and anhedonia (item 1) concurrently scored above 50 on the corresponding M3VAS item (28 and 27%, respectively) than for suicidality (3.4%). Such inferences are limited given the exclusion of higher-risk patients in this study, and it would be imperative to further explore how those with a clinically verified risk of suicide respond on M3VAS. Future utilisation of M3VAS in both research and clinical practice could potentially be developed to consider the two core symptoms as a distinct measure, themselves forming a more focused measure of depression severity,^42^ and incorporation of the suicidality item could then find clinical relevance to either augment the global view of depression severity or aid more specifically in risk assessment. Determining whether individual item scores change independently or at different speeds may also be of interest, considering the possibility of preferential symptom targeting by a particular drug class or therapy modality.^43^

The structural validity of PHQ-9 has been extensively explored elsewhere^44^ and was included here for illustrative contrast against the ultra-short measures, with our results somewhat mirroring the inconsistency in dimensionality and item-to-factor loadings that has been seen across samples. Alpha remained in the excellent range of 0.91–0.93, indicating a high degree of interrelatedness of the items within PHQ-9, a feature that is likely to be raised in longer scales assessing the same construct.^33^ PHQ-9 displayed a strong single factor at w0 and w4, and a two-factor structure at w2 – the latter in accord with several large-scale evaluations that distinguish apparent ‘affective’ and ‘somatic’ (sleep and appetite difficulties, fatigue) symptom groups.^45,46^ The utility of parsing out such clusters/symptomatic phenotypes within depression has prompted significant research investment, with an emerging suggestion that such clusters may map to the Research Domain Criteria matrix of mental disorders,^47^ and that their relative predominance in certain groups can impact on chronicity,^48^ treatment response^49^ and consequent physical health risks.^50^

This study confirmed strong convergence of M3VAS with PHQ-9 at all time points, with correlation values appearing higher (*r* = 0.86–0.91 in this sample) than in a previous study comparing against QIDS-SR-16^23^ (*r* = 0.72). While it would be premature to draw firm conclusions about the relative convergence of M3VAS between these two gold-standard PROMs, this finding could reflect a weaker validity of QIDS-SR-16, as recently proposed.^4^ Individual item correlations between M3VAS and corresponding symptoms on PHQ were also generally high (Pearson’s *r* = 0.70–0.92).

Different strands within the psychometric literature endorse varied approaches to capturing responsiveness in an outcome measure,^36,51^ defined as an ‘instrument’s ability to detect change over time in the construct to be measured’. We have performed a broad evaluation in this study. Despite the study’s short duration and lack of intervention, we hypothesised that a degree of symptom fluctuation, including naturalistic treatment responses, would be expected (at least in people with affective disorder). Appropriate longitudinal validity for M3VAS has been inferred in this study from both the strong correlation of score changes over the 4-week study interval with those seen on PHQ-9 (as the gold-standard, criterion approach), and a significant effect of time on scores in the depressed group as assessed by ANOVA and moderate effect size (Cohen’s *d*). Such effects were likewise demonstrated on PHQ-9 as the reference standard (construct approach) to a higher magnitude than for M3VAS in this sample. With limited published data on longitudinal comparison of VAS and Likert scales for depression, our findings therefore currently caution against any tentative expectations of M3VAS offering favourable sensitivity to change over established gold-standard Likert scales. However, longer studies with standardised interventions would clearly be optimal for examining PROMs’ relative sensitivity as end-points for capturing clinically relevant treatment responses (including remission), and this is the proposed next step for evaluation of M3VAS. Hypotheses that VAS may offer superiority in that regard have arisen from comparisons with scales assessing other affective constructs – such as pain^52^ and general well-being^15^ – and continue to merit exploration, particularly given their relative ease of administration.

Score change correlation between M3VAS and PHQ-9 did appear slightly weaker in healthy controls, with the reduced score variability in this group being one possible contributor. However, the small numbers in this group challenge interpretation. For complete longitudinal validation of M3VAS, exclusion of measurement error on repeat testing in a larger healthy/euthymic population should be considered.^53^ The ability of M3VAS to detect new episodes of depression in these populations may also be a distinct longitudinal measurement property from capturing the degree of treatment response, and would merit further exploration.

### Limitations

As mentioned above, expectations of symptom change in this secondary data analysis from the RHAPSODY study were limited by relatively short follow-up, and also by varied treatment status, introducing additional heterogeneity in expected effects. The attrition was also noteworthy (72 and 74% completing w4 PHQ-9 and M3VAS, respectively). While reasons for study exit are not formally captured, one could hypothesise that patients who were more depressed were more likely to find the intensive battery of cognitive and speech tasks (as part of the primary study) intolerably burdensome. Although this could feasibly overestimate the magnitude of symptom change, it would not be deemed a marked source of bias for within-subject comparisons (i.e. correlation) of the different scales, including over time.

Our secondary analysis was not powered *a priori* to detect specified clinical outcomes or psychometric properties. In reference to COSMIN best practice guidelines,^26^ both the pooled and affective disorder groups appear adequate for the criterion (correlation) approach which, coupled with the strength of correlation, would be considered appropriate preliminary assessment of longitudinal validity. As per COSMIN, our sample would also be considered appropriate to evaluate responsiveness through a construct approach in testing hypotheses of group differences in change over time (group × time interaction), and an effect of time in the pooled sample (the latter not reaching statistical significance in our study). There is, however, some doubt as to the external validity in extrapolating certain group-specific findings, namely the within-group effect of time (which was compounded by the attrition observed) and the correlations in the smaller control group. Thus, we emphasise the need for larger-scale testing, both for depressed cohorts under controlled treatment conditions and for further validation in euthymic/healthy samples.

## Supporting information

Silman et al. supplementary material Silman et al. supplementary material

## Data Availability

The data that support the findings of this study are subject to request from the corresponding author, R.S. The data are not publicly available due to ongoing analysis of the primary outcomes for the RHAPSODY study (including speech phenotyping) by the study sponsor. All analysis and research materials associated with this manuscript can be provided on reasonable request. M3VAS is freely available for the use of any non-profit organisation (including academics and clinicians), accessed via https://www.kcl.ac.uk/research/m3vas; for-profit organisations may need to purchase a licence. Please contact the corresponding author for access.

## Supplementary material

The supplementary material is available online at https://doi.org/10.1192/bjo.2025.10932
