# Supplementary material for: Longitudinal validation of the Maudsley 3-item visual analogue scale (M3VAS): a new, brief, patient-reported outcome measure of depression
Source: BJPsych Open. 2025 Dec 22;12(1):e23. doi: 10.1192/bjo.2025.10932 (PMC12724119; doi:10.1192/bjo.2025.10932)
Supplement: Silman et al. supplementary material [file S2056472425109320sup001.docx]

**Longitudinal validation of The Maudsley 3-item visual analogue scale (M3VAS): a new brief measure for patient monitoring of depression**

Supplementary information

**Table S1:** Discrepancies from pre-print protocol:

**doi:** <https://doi.org/10.1101/2023.06.26.23291655>

| Protocol section | Revisions from pre-print made |
| --- | --- |
| Hypotheses & objectives: | reconceptualised (as opposed to published content being inaccurate reflection of the work). Longitudinal validity was clarified to reflect assessment of responsiveness as defined by COSMIN criteria – correlation of score changes as well as construct approaches (effect of time). Determining stability of M3VAS internal structure (factorial validity and internal consistency) was also added as an element of longitudinal assessment. |
| Participants | affective disorders group n = 50 consisting of Major Depressive Disorder, n = 24 (not 25), and Bipolar Affective disorder, n = 26 (not 25). Healthy controls n = 24 (not 26) |
| Analyses | - Additional analyses splitting PHQ-9 into PHQ-3 which contains directly matched items to M3VAS and PHQ-6 which contains all other items. - Addition of longitudinal assessment of internal structure – factor analysis, internal consistency - a Bland-Altman analysis was not performed as deemed not relevant to longitudinal change over time |

**Table S2.** Factor analysis of the three symptom measures: M3VAS, PHQ-3 and PHQ-9 repeated at study timepoints baseline, week 2 and week 4.

| **M3VAS - week 0** | | | | | | |
| --- | --- | --- | --- | --- | --- | --- |
| KMO | 0.599 | bartlett's chi sq | 145.063 | p | <0.001 |  |
| Component | Initial Eigenvalues | |  | Item loadings | Component | |
|  | Total | % of Variance | Cumulative % |  | 1 | n/a |
| 1 | 2.263 | 75.429 | 75.429 | W0\| M3VAS mood | 0.949 |  |
| 2 | 0.648 | 21.612 | 97.041 | W0\| M3VAS interest | 0.931 |  |
| 3 | 0.089 | 2.959 | 100.000 | W0\| M3VAS suicide | 0.704 |  |
| **M3VAS - week 2** | | | | | | |
| KMO | 0.633 | bartlett's chi sq | 96.963 | p | <0.001 |  |
| Component | Initial Eigenvalues | |  | Item loadings | Component | |
|  | Total | % of Variance | Cumulative % |  | 1 | n/a |
| 1 | 2.229 | 74.287 | 74.287 | W2\| M3VAS mood | 0.925 |  |
| 2 | 0.614 | 20.464 | 94.751 | W2\| M3VAS interest | 0.917 |  |
| 3 | 0.157 | 5.249 | 100.000 | W2\| M3VAS suicide | 0.730 |  |
| **M3VAS - week 4** | | | | | | |
| KMO | 0.632 | bartlett's chi sq | 56.561 | p | <0.001 |  |
| Component | Initial Eigenvalues | |  | Item loadings | Component | |
|  | Total | % of Variance | Cumulative % |  | 1 | n/a |
| 1 | 2.134 | 71.129 | 71.129 | W4\| M3VAS mood | 0.910 |  |
| 2 | 0.622 | 20.741 | 91.869 | W4\| M3VAS interest | 0.872 |  |
| 3 | 0.244 | 8.131 | 100.000 | W4\| M3VAS suicide | 0.738 |  |
| **PHQ3 - week 0** | | | | | | |
| KMO | 0.618 | bartlett's chi sq | 102.755 | p | <0.001 |  |
| Component | Initial Eigenvalues | |  | Item loadings | Component | |
|  | Total | % of Variance | Cumulative % |  | 1 | n/a |
| 1 | 2.219 | 73.964 | 73.964 | W0\| Low mood | 0.932 |  |
| 2 | 0.605 | 20.180 | 94.144 | W0\| Anhedonia | 0.891 |  |
| 3 | 0.176 | 5.856 | 100.000 | W0\| Suicidality | 0.746 |  |
| **PHQ3 - week 2** | | | | | | |
| KMO | 0.648 | bartlett's chi sq | 101.826 | p | <0.001 |  |
| Component | Initial Eigenvalues | |  | Item loadings | Component | |
|  | Total | % of Variance | Cumulative % |  | 1 | n/a |
| 1 | 2.295 | 76.503 | 76.503 | W2\| Low mood | 0.933 |  |
| 2 | 0.546 | 18.187 | 94.690 | W2\| Anhedonia | 0.907 |  |
| 3 | 0.159 | 5.310 | 100.000 | W2\| Suicidality | 0.776 |  |
| **PHQ3 - week 4** | | | | | | |
| KMO | 0.699 | bartlett's chi sq | 73.779 | p | <0.001 |  |
| Component | Initial Eigenvalues | |  | Item loadings | Component | |
|  | Total | % of Variance | Cumulative % |  | 1 | n/a |
| 1 | 2.328 | 77.589 | 77.589 | W4\| Low mood | 0.911 |  |
| 2 | 0.456 | 15.191 | 92.780 | W4\| Anhedonia | 0.906 |  |
| 3 | 0.217 | 7.220 | 100.000 | W4\| Suicidality | 0.823 |  |
| **PHQ-9 - week 0** | | | | | | |
| KMO | 0.904 | bartlett's chi sq | 511.588 | p | <0.001 |  |
| Total Variance Explained | | |  |  |  |  |
| Component | Initial Eigenvalues | |  | Item loadings | Component | |
|  | Total | % of Variance | Cumulative % |  | 1 | n/a |
| 1 | 5.913 | 65.697 | 65.697 | W0\| Anhedonia | 0.854 |  |
| 2 | 0.827 | 9.187 | 74.883 | W0\| Low mood | 0.914 |  |
| 3 | 0.613 | 6.808 | 81.691 | W0\| Sleep | 0.877 |  |
| 4 | 0.582 | 6.470 | 88.161 | W0\| Low energy | 0.891 |  |
| 5 | 0.300 | 3.337 | 91.498 | W0\| Appetite | 0.738 |  |
| 6 | 0.250 | 2.782 | 94.280 | W0\| Guilt | 0.786 |  |
| 7 | 0.215 | 2.384 | 96.664 | W0\| Concentration | 0.816 |  |
| 8 | 0.177 | 1.971 | 98.634 | W0\| Psychomotor retardation | 0.735 |  |
| 9 | 0.123 | 1.366 | 100.000 | W0\| Suicidality | 0.644 |  |
| **PHQ-9 - week 2** | | | | | | |
| KMO | 0.977 | bartlett's chi sq | 355.578 | p | <0.001 |  |
| Component | Initial Eigenvalues | |  | Item loadings | Component | |
|  | Total | % of Variance | Cumulative % |  | 1 | 2 |
| 1 | 5.298 | 58.864 | 58.864 | W2\| Anhedonia | 0.634 | 0.347 |
| 2 | 1.041 | 11.564 | 70.428 | W2\| Low mood | 0.726 | 0.238 |
| 3 | 0.652 | 7.241 | 77.669 | W2\| Sleep | -0.094 | 0.956 |
| 4 | 0.633 | 7.035 | 84.704 | W2\| Low energy | -0.117 | 0.942 |
| 5 | 0.431 | 4.787 | 89.491 | W2\| Appetite | -0.025 | 0.770 |
| 6 | 0.320 | 3.558 | 93.049 | W2\| Guilt | 0.474 | 0.412 |
| 7 | 0.287 | 3.191 | 96.240 | W2\| Concentration | 0.521 | 0.348 |
| 8 | 0.200 | 2.221 | 98.461 | W2\| Psychomotor retardation | 0.779 | -0.007 |
| 9 | 0.139 | 1.539 | 100.000 | W2\| Suicidality | 1.035 | -0.362 |
|  | Rotation Sums of Squared Loadingsa | | | Total | 4.569 | 4.491 |
| **PHQ-9 - week 4** | | | | | | |
| KMO | 0.891 | bartlett's chi sq | 321.246 | p | <0.001 |  |
| Component | Initial Eigenvalues | |  | Item loadings | Component | |
|  | Total | % of Variance | Cumulative % |  | 1 | n/a |
| 1 | 5.761 | 64.007 | 64.007 | W4\| Anhedonia | 0.870 |  |
| 2 | 0.757 | 8.414 | 72.420 | W4\| Low mood | 0.923 |  |
| 3 | 0.727 | 8.073 | 80.493 | W4\| Sleep | 0.786 |  |
| 4 | 0.450 | 4.998 | 85.491 | W4\| Low energy | 0.826 |  |
| 5 | 0.422 | 4.691 | 90.182 | W4\| Appetite | 0.773 |  |
| 6 | 0.345 | 3.835 | 94.017 | W4\| Guilt | 0.817 |  |
| 7 | 0.234 | 2.598 | 96.615 | W4\| Concentration | 0.847 |  |
| 8 | 0.175 | 1.946 | 98.561 | W4\| Psychomotor retardation | 0.658 |  |
| 9 | 0.130 | 1.439 | 100.000 | W4\| Suicidality | 0.661 |  |

*Factor analysis by Principal axis factoring was evaluated at the three study timepoints. M3VAS, PHQ-9 and its derivative PHQ-3 containing only matched items to the M3VAS. Abbreviations: M3VAS = Maudsley 3-item visual analogue scale for depression; PHQ = Patient Health Questionnaire. KMO = Kaiser-Meyer-Olkin. Interpretation: KMO > 0.6 and a significant Bartlett’s Test of Sphericity (p < .001) indicate suitability to perform principal axis factoring. Eigenvalues (λ) > 1.0 indicates number of factors to extract. Items with factor loadings ≤ 0.5 are problematic regarding factor structure*

**Table S3:** Internal consistency / reliability for M3VAS, PHQ-9 and its derivative PHQ-3 containing only matched items to the M3VAS, at the three measurement timepoints – Cronbach’s alpha (α), Alpha if item deleted (AID) and corrected item-total-correlation (ITC)

|  | wk0 | | | wk 2 | | | wk 4 | | |
| --- | --- | --- | --- | --- | --- | --- | --- | --- | --- |
|  | Overall α | C- ITC | AID | Overall α | C- ITC | AID | Overall α | C- ITC | AID |
| ***M3VAS*** | 0.83 |  |  | 0.83 |  |  | 0.78 |  |  |
| Low mood |  | 0.88 | 0.56 |  | 0.80 | 0.64 |  | 0.78 | 0.51 |
| Anhedonia |  | 0.84 | 0.62 |  | 0.78 | 0.66 |  | 0.72 | 0.59 |
| Suicidality |  | 0.48 | 0.95 |  | 0.51 | 0.91 |  | 0.50 | 0.85 |
| ***PHQ-3*** | 0.82 |  |  | 0.85 |  |  | 0.84 |  |  |
| Low mood |  | 0.83 | 0.59 |  | 0.83 | 0.67 |  | 0.80 | 0.67 |
| Anhedonia |  | 0.74 | 0.69 |  | 0.77 | 0.73 |  | 0.79 | 0.68 |
| Suicidality |  | 0.52 | 0.90 |  | 0.57 | 0.91 |  | 0.64 | 0.88 |
| ***PHQ-9*** | 0.93 |  |  | 0.91 |  |  | 0.93 |  |  |
| Low mood |  | 0.88 | 0.92 |  | 0.82 | 0.89 |  | 0.89 | 0.91 |
| Anhedonia |  | 0.80 | 0.92 |  | 0.84 | 0.89 |  | 0.82 | 0.91 |
| Suicidality |  | 0.58 | 0.94 |  | 0.53 | 0.91 |  | 0.58 | 0.93 |
| Sleep |  | 0.84 | 0.92 |  | 0.71 | 0.90 |  | 0.73 | 0.92 |
| Low energy |  | 0.85 | 0.92 |  | 0.67 | 0.90 |  | 0.77 | 0.92 |
| Appetite |  | 0.67 | 0.93 |  | 0.60 | 0.91 |  | 0.71 | 0.92 |
| Guilt |  | 0.72 | 0.93 |  | 0.73 | 0.90 |  | 0.75 | 0.92 |
| Concentration |  | 0.76 | 0.93 |  | 0.72 | 0.90 |  | 0.80 | 0.91 |
| Psychomotor retardation |  | 0.67 | 0.93 |  | 0.62 | 0.90 |  | 0.58 | 0.93 |

**Table S4:** Convergent validity (Pearson’s correlation r) between individual mood, anhedonia, and suicidality items across scales

|  |  | M3VAS item | | |
| --- | --- | --- | --- | --- |
|  | PHQ-9 item | low mood | anhedonia | suicidality |
| Baseline | Low mood | **0.84** | **0.77** | **0.48** |
|  | Anhedonia | **0.82** | **0.86** | **0.42** |
|  | Suicidality | **0.50** | **0.47** | **0.76** |
| Week 2 | Low mood | **0.79** | **0.73** | **0.47** |
|  | Anhedonia | **0.81** | **0.77** | **0.43** |
|  | Suicidality | **0.48** | **0.48** | **0.85** |
| Week 4 | Low mood | **0.85** | **0.65** | **0.48** |
|  | Anhedonia | **0.74** | **0.71** | **0.52** |
|  | Suicidality | **0.49** | **0.47** | **0.70** |
| Overall | Low mood | **0.82** | **0.72** | **0.46** |
|  | Anhedonia | **0.79** | **0.79** | **0.43** |
|  | Suicidality | **0.50** | **0.47** | **0.78** |

All values were statistically significant (p < 0.001). Shaded boxes denote correlation between the corresponding items.

**Table S5:** *Analysis of variance (ANOVA) examining effect of time, interaction of time & group as within-subjects factors; and between subjects effect of group: M3VAS, PHQ-9 & PHQ-3*

|  | M3VAS | | PHQ-9 | | PHQ-3 | |
| --- | --- | --- | --- | --- | --- | --- |
| Effect of time: |  |  |  |  |  |  |
| combined | W | p | W | p | W | p |
|  | .999 | .966 | .844 | *.016* | .870 | *.033* |
|  | F | p | F | p | F | p |
|  | 3.039 | .052 | 10.073^ | **<0.001** | 6.285^ | **.004** |
| AD | W | p | W | p | W | p |
|  | .977 | .684 | .874 | .132 | .894 | .187 |
|  | F | p | F | p | F | p |
|  | 4.942 | **0.010** | 12.505 | **<0.001** | 8.527 | **<0.001** |
| HC | W | p | W | p | W | p |
|  | .500 | *0.002* | .985 | .883 | .742 | .079 |
|  | F | p | F | p | F | P |
|  | .424^ | .578 | .082 | .922 | .376 | .689 |
| Interaction time & group | W | p | W | p | W | p |
|  | .992 | .821 | .895 | .069 | .915 | .120 |
|  | F | P | F | P | F | p |
|  | 3.591 | **.031** | 6.140 | **.003** | 5.641 | **.005** |
| Between subjects effect of group | F | p | F | p | F | p |
|  | 132.4 | **<0.001** | 132.7 | **<0.001** | 96.84 | **<0.001** |

*Abbreviations: M3VAS = Maudsley 3-item visual analogue scale for depression; PHQ = Patient Health Questionnaire. Key: W = Mauchly's sphericity test to examine equal variances of within-subject conditions. P < 0.05 is indicated by italics which violates the assumption, such that Greenhouse-Geisser Correction (^) is applied to the ANOVA (F). bold typeface for associated p value (<0.05) to F indicates significant effect / interaction of variables on ANOVA.*
